# Simultaneous Enhancement of Mildew Resistance and Dimensional Stability of Bamboo with a Facile One-Step In Situ Growth of ZnO/TA/Ag Composites

**DOI:** 10.3390/molecules31101737

**Published:** 2026-05-19

**Authors:** Juan Xu, Jinju Ma, Lanxiang Liu, Baoshan Tang, Hong Zhang, Wenwen Zhang, Zhengjun Shi

**Affiliations:** 1 Key Laboratory of State Forestry and Grassland Administration on Highly-Efficient Utilization of Forestry Biomass Resources in Southwest China, Southwest Forestry University, Kunming 650224, China; xujuan89@hotmail.com; 2 Institute of Highland Forest Science, Chinese Academy of Forestry, Kunming 650233, China; 3 Yunnan Key Laboratory of Breeding and Utilization of Resource Insects, Kunming 650224, China; 4 School of Pharmacy, Xinyang Agriculture and Forestry University, Xinyang 464000, China

**Keywords:** bamboo, ZnO/TA/Ag composites, *Aspergillus niger*, *Penicillium citrinum*, antimildew resistance, dimensional stability

## Abstract

Bamboo is a renewable and fast-growing biomass resource with limited utilization and service life owing to its susceptibility to mold. Conventional nano-modification methods, particularly two-step approaches, are limited by weak interfacial bonding between nanoparticles and the bamboo substrate, complex processing, and an inability to simultaneously enhance antimildew performance and dimensional stability. To address these limitations, we developed a one-step hydrothermal method involving the use of tannic acid (TA) for in situ fabrication of ZnO/TA/Ag composite particles on bamboo surfaces. Process parameters were optimized to 100 °C, 10 h, and a zinc acetate-to-tannic acid molar ratio of 20:1. The modified bamboo was characterized using Fourier-transform infrared spectroscopy, X-ray photoelectron spectroscopy, X-ray diffraction, scanning electron microscopy coupled with energy-dispersive spectroscopy, and thermogravimetric analysis. We demonstrated that ZnO/TA/Ag composite particles were successfully loaded onto the bamboo surface, thus improving the all-around performance of the bamboo simultaneously. Antimildew activity against *Aspergillus niger* and *Penicillium citrinum* increased from grade 4 in untreated bamboo to grades 1 and 0, respectively; water absorption decreased by 52.85%, and anti-swelling efficiency reached 30.41%, indicating improved mold resistance and dimensional stability. Thus, our technique could serve as a green and efficient one-step in situ modification strategy for high-performance functionalization of bamboo, making it suitable for applications in humid outdoor and indoor environments.

## 1. Introduction

With the growing emphasis on green and low-carbon development in materials science, the development of biomass materials that combine high durability with environmental compatibility has become a key research direction [1,2,3,4]. Bamboo is increasingly recognized as a promising alternative to conventional materials such as timber, plastics, and steel owing to its rapid renewability, high carbon sequestration potential, and favorable mechanical properties [5,6]. However, practical engineering applications of bamboo are constrained by inherent limitations associated with hierarchical porous anatomy and pronounced hydrophilicity [7,8]. First, the abundance of non-structural carbohydrates (such as starch and sugar) stored in its cells provides a nutrient-rich substrate for fungal colonization and subsequent biotic degradation. Second, poor dimensional stability, which results in warping and cracking, affects the long-term performance of bamboo products in open or humid environments.

Strategies for improving mildew resistance include physical treatment, chemical treatment, and imprinting with an antimildew agent [9,10,11], as well as thermal treatment and resin impregnation to reduce hydrophilicity and improve dimensional stability [8,12]. However, such approaches often compromise mechanical properties, and resin systems may release volatile organic compounds (VOCs), which raises environmental and health issues. Chemical preservatives, such as those based on copper compounds or organic fungicides, exhibit strong antimildew efficacy but show rapid loss of active ingredients, potential toxicity, and limited improvement in dimensional stability [7,13].

Recent advances in nanotechnology provide opportunities for the multifunctional enhancements of bamboo. Various inorganic nanoparticles, including ZnO, TiO_2_, Cu, and Ag, as well as alloys and composites, have been investigated for their antimildew activity, antibacterial properties, UV resistance, and hydrophobicity [14,15,16,17]. Wang et al. developed a bilayer nano-SiO_2_ coating comprising a dense moisture-barrier underlayer and a fluorinated nanoporous top layer to achieve antimildew performance [18]. Ag-Cu composite nanoparticles applied as coatings helped achieve 100% antibacterial activity within 1 min at concentrations as low as 10 ppm and showed improved durability through reduced particle dissolution [19]. Via the photocatalytic generation of reactive oxygen species by ZnO and TiO_2_, Wang et al. developed bamboo plywood with inherent antimildew and antibacterial properties [20]. Tang et al. constructed a biomimetic coating using TiO_2_ nanoparticles, methyl-trimethoxysilane, and paraffin, and it demonstrated excellent antimildew and self-cleaning performance [21].

In situ synthesis directly triggers the nucleation and growth of functional nanoparticles on a substrate, particularly effective for securely anchoring particles [22,23,24,25,26]. Biomass-derived molecules have recently gained prominence as environmentally friendly reaction media and agents for directing the structure of sustainable nanosynthesis [27,28,29]. Tannic acid (TA), a natural polyphenol, contains abundant catechol and pyrogallol groups. These moieties confer strong metal ion complexation and reduction capabilities [28,30], and they also promote tight binding to bamboo lignocellulose via hydrogen bonds, hydrophobic interactions, and π-π stacking [31,32]. Consequently, TA functions both as a green reducing agent and stabilizer for silver nanoparticle synthesis and as an interfacial bridging molecule that strengthens adhesion between the nanocoating and the bamboo surface.

In this study, we used tannic acid as a reducing and chelating agent to reduce Ag^+^, complex Zn^2+^, and form strong hydrogen-bonded attachments to active groups on the bamboo surface. We developed an innovative TA-mediated one-step hydrothermal method to assemble ZnO/TA/Ag ternary composite particles on bamboo in situ. The composite is designed to synergistically improve mildew resistance and dimensional stability via three mechanisms: the physical barrier provided by ZnO, interfacial reinforcement afforded by TA, and the chemical antibacterial action of Ag nanoparticles. We systematically examined key process parameters, including reaction temperature, reaction time, and the mass concentration ratio of zinc acetate to TA, to determine their effects on TA and composite particle loading and identified optimal conditions. We characterized the surface micromorphology, crystal structure, and surface chemistry of bamboo before and after modification. Finally, by combining mildew resistance testing with water absorption and volume expansion analyses, we comprehensively evaluated the mildew resistance and dimensional stability of the modified bamboo, thereby confirming the successful construction of ZnO/TA/Ag composite particles and their effects on the properties of bamboo.

## 2. Results and Discussion

### 2.1. Effects of Process Conditions on Weight Gain Rate and TA Loading on the Bamboo Surface

**Figure 1:**
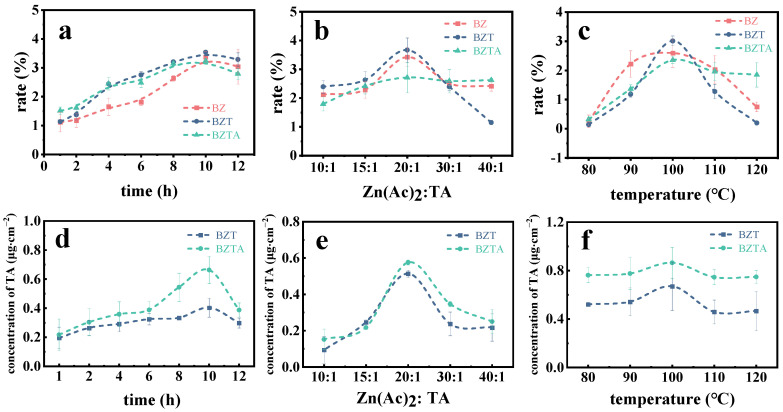
The influence of hydrothermal reaction time ((**a**): weight rate; (**d**): concentration of TA), the mass concentration ratio of zinc acetate to TA ((**b**): weight rate; (**e**): concentration of TA) and temperature ((**c**): weight rate; (**f**): concentration of TA) on the weight rate of samples and the TA content on the surface of bamboo samples.

To investigate the influence of reaction time on the quality changes of bamboo and the content of tannic acid on its surface, the reaction temperature (100 °C) and the mass concentration ratio of zinc acetate to tannic acid (20:1) were kept constant. The reaction times were set at 1 h, 2 h, 4 h, 6 h, 8 h, 10 h, and 12 h, respectively. As shown in Figure 1a,d, with an increase in the reaction time, the weight rates of the BZ, BZT, and BZTA bamboo samples, as well as the TA content on the surface of the BZT and BZTA samples, all showed an initial increase followed by a decrease. At a reaction time of 10 h, both the weight rates and TA contents of the bamboo samples reached their maximum values. The weight rates of BZ, BZT, and BZTA were 3.37%, 3.54%, and 3.24%, and the TA contents of BZT and BZTA were 0.40 μg/cm^2^ and 0.66 μg/cm^2^. This finding suggests that the BZTA sample exhibits a greater capacity for TA loading. When the hydrothermal reaction time was extended to 12 h, both the weight rate (e.g., BZTA decreased to 2.79%) and the tannic acid (TA) content of the samples declined. This decrease could be attributed to two factors. First, prolonged reaction time promoted the aggregation of ZnO, ZnO-TA, and ZnO-TA-Ag composite particles, causing small particles to gradually aggregate and deposit as larger agglomerates (Figure S1, Supporting Information). This aggregation reduced specific surface area and binding sites, leading to the removal of some particles from bamboo. Second, for prolonged hydrothermal conditions, the catechol groups of TA may have been oxidized to quinones (Figures S2 and S3 and Table S1), compromising their coordination with Zn^2+^. Hence, a 10 h reaction time was chosen for future experiments.

At temperatures below 100 °C and with a treatment duration of 10 h, the effect of the mass concentration ratio of zinc acetate to TA on the samples’ weight rate and surface TA content was investigated. The results are illustrated in Figure 1b,e. As the mass concentration ratio increased, both the weight rate and surface TA content of the samples initially rose before subsequently declining. Specifically, when the mass concentration ratio of zinc acetate to TA was raised from 10:1 to 20:1, the weight rate and surface TA content of the samples increased correspondingly. Sample BZT exhibited the largest increase in weight rate, from 1.15% to 3.67%, followed by sample BZ. By contrast, sample BZTA showed a modest increase of only 0.92%. These results indicate that, within this mass concentration ratio range, Zn^2+^ effectively coordinates with TA phenolic hydroxyl groups to form a stable ZnO-TA complex, while TA more effectively anchors to the bamboo surface and functions as a bridge. However, in the BZTA sample, the introduction of Ag^+^ occupies some of the phenolic hydroxyl sites of TA, which partially weakens its bridging efficiency, resulting in a lower increase in weight rate. When the mass concentration ratio exceeded 20:1, both the WPG and the TA content began to decline, likely because an excessively high Zn^2+^ concentration promotes rapid agglomeration of ZnO particles, reducing their effective specific surface area and the number of active sites. At a mass concentration ratio of 20:1, the TA contents of BZT and BZTA reached their maximum values of 0.51 μg/cm^2^ and 0.58 μg/cm^2^, respectively. Therefore, a mass concentration ratio of zinc acetate to TA of 20:1 was selected for subsequent experiments.

Figure 1c,f illustrate the effect of reaction temperature (80–120 °C) on weight rate and TA loading under fixed conditions of Zn(AC)_2_:TA ratio of 20:1 and a reaction time of 10 h. Both parameters initially increased and then decreased with rising temperature. Maximum TA loadings on BZT and BZTA were achieved at 100 °C, reaching 0.67 μg/cm^2^ and 0.86 μg/cm^2^, respectively. Within 80–90 °C, TA loading showed minimal variation. However, from 90 °C to 100 °C, TA loading increased by 23.67% and 11.53%, respectively. Further temperature increase reduced both parameters, which can be attributed to the oxidation or thermal decomposition of TA. Across the tested temperature range, the TA content of the BZTA sample consistently exceeded that of BZT. Consequently, a reaction temperature of 100 °C was selected based on these findings.

### 2.2. Structural Analysis of ZnO, TA, and Ag Incorporation

**Figure 2:**
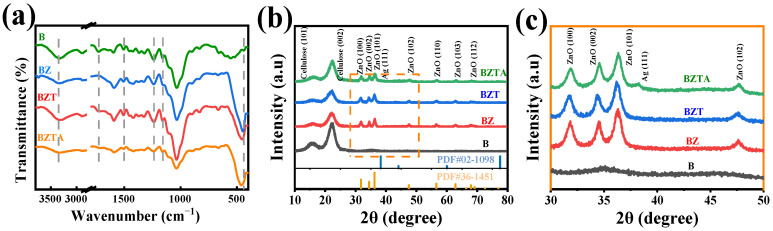
FTIR spectrogram (**a**) and XRD (**b**,**c**) of B, BZ, BZT, and BZTA (the orange box in (**b**) marks the region that is magnified in (**c**)).

ATR-FTIR analysis was utilized to examine functional groups in the B, BZ, BZT, and BZTA samples, with the corresponding spectra depicted in Figure 2a. In sample B, the absorption peak at 3328 cm^−1^ corresponds to O-H stretching, and the peak at 2890 cm^−1^ is attributed to C-H stretching vibrations. The bands in proximity to 1590 cm^−1^ and 1510 cm^−1^ are linked to the aromatic rings in lignin, and the signal at 1423 cm^−1^ pertains to the H-C-H in-plane bending vibration of cellulose [33]. The absorption peak around 1050 cm^−1^ is associated with the stretching vibration of the ether bond (C-O-C), whereas the band at 890 cm^−1^ is connected to the β-glycosidic links between xylose units [30,34].

Notably, sample B exhibited a prominent absorption peak at 1728 cm^−1^, attributed to the ester bonds involving lignin or lignin–carbohydrate interactions [35]. In contrast, the peak observed in BZ was weak and nearly absent, indicating that the hydrothermal treatment of bamboo influenced the ester linkages. However, following the introduction of TA, both BZT and BZTA displayed significant C=O stretching vibrations at 1728 cm^−1^, with the C-O stretching peak being at 1160 cm^−1^, characteristic of the ester bonds present in TA molecules [31]. A new absorption peak at 454 cm^−1^ appears in the BZ sample and is assignable to the Zn-O stretching vibration [36,37], indicating successful loading of ZnO onto the bamboo surface. In the BZT and BZTA samples, where TA alone and TA with Ag were introduced, the Zn-O absorption peak persists but is slightly shifted. This shift likely reflects hydrogen-bonding or coordination interactions between TA and ZnO, or between Ag^+^ and TA/ZnO, which alter the Zn-O vibrational frequency.

The crystal structures of the B, BZ, BZT, and BZTA samples were examined by XRD, and the results are shown in Figure 2b,c. The XRD pattern of the B sample shows two diffraction peaks at 16.0° and 22.2°, which are assigned to the crystalline regions of bamboo cellulose [38]. After treatment, the BZ, BZT, and BZTA samples all display identical diffraction peaks at 31.8°, 34.5°, 36.3°, 47.6°, 56.5°, 62.9°, and 68.0°. These peaks correspond to the (100), (002), (101), (102), (110), (103), and (112) crystal planes of hexagonal wurtzite ZnO, respectively [39]. The peak positions match PDF card #36-1451 for wurtzite-type ZnO, indicating successful loading of ZnO onto the BZ, BZT, and BZTA surfaces and showing that TA introduction did not alter the ZnO crystal structure. Furthermore, the BZTA pattern contains, in addition to the bamboo cellulose and ZnO peaks, a new diffraction peak at 38.2°, which corresponds to the (111) plane of Ag(0) [40]. The absence of Ag(I) characteristic peaks indicates that the introduced Ag^+^ was reduced to elemental Ag by TA.

### 2.3. Surface Chemical Composition Analysis

**Figure 3:**
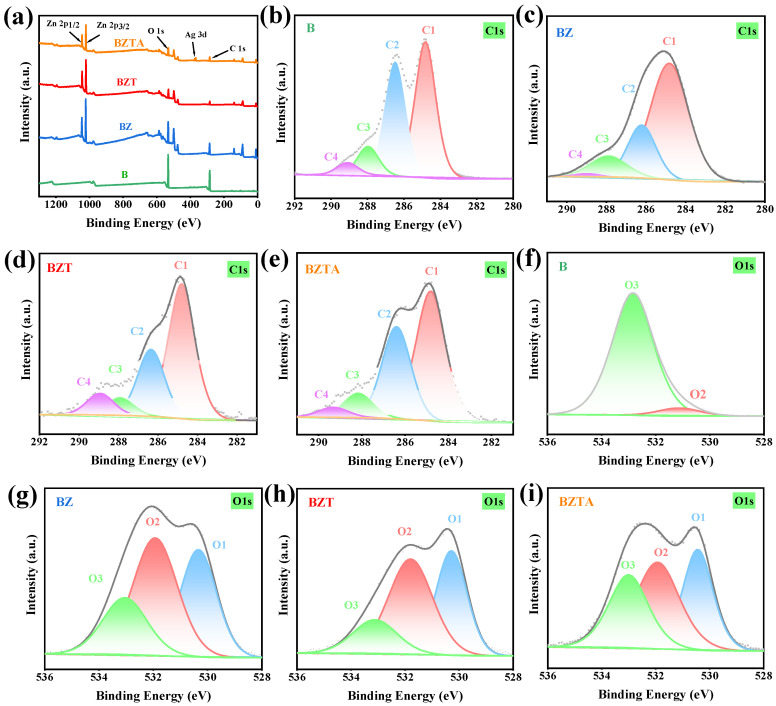
XPS spectrograms of B, BZ, BZT, and BZTA (survey (**a**), C1s (**b**–**e**), O 1s (**f**–**i**)).

XPS was employed to characterize the chemical composition of the B, BZ, BZT, and BZTA samples. The full spectrum (Figure 3a) shows that the B sample contains primarily C and O. The BZ and BZT samples contain C, O, and Zn, while the BZTA sample contains C, O, Zn, and Ag, in agreement with the SEM-EDS analysis.

**Figure 4:**
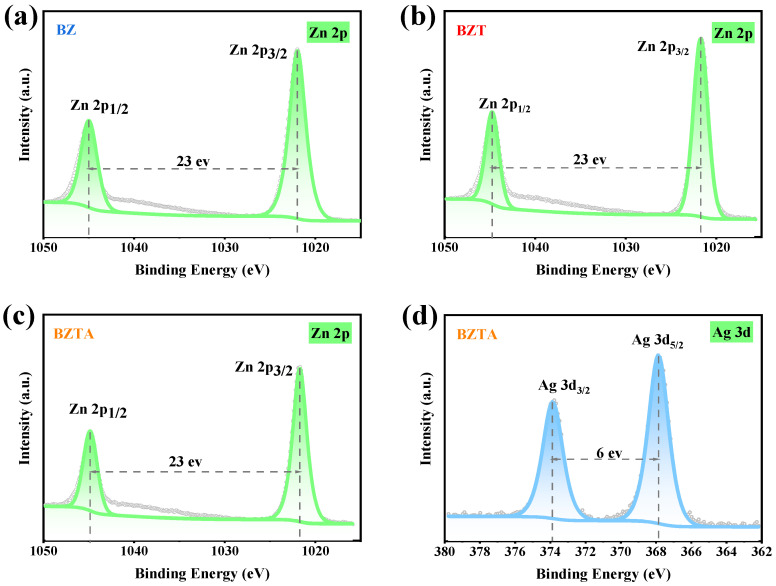
XPS spectrograms of B, BZ, BZT, BZTA, Zn 2p (**a**,**b**), and Ag 3d (**c**,**d**).

The C1s spectra of B, BZ, BZT, and BZTA were categorized into four components: C1 (C-C or C-H), C2 (C-OH), C3 (O-C-O or C=O), and C4 (O-C=O) [41]. After introducing zinc ions, interaction between ZnO and hydroxyl groups on the bamboo surface caused shifts in the binding energies of the C2, C3, and C4 components in the BZ sample from 286.47 eV, 287.96 eV, and 289.09 eV to 286.19 eV, 287.88 eV, and 288.96 eV, respectively. The subsequent addition of TA further shifted these binding energies to 286.35 eV, 287.92 eV, and 288.93 eV, which is attributed to the coordination between the phenolic hydroxyl groups of TA and zinc ions (Figure 4d). As summarized in Table 1, the relative content of C1 increased from 47.15% in B to 65.16% in BZ, suggesting that oxygen-containing functional groups (e.g., -OH and -CHO in lignin and cellulose) were partially covered during treatment, thereby increasing the proportion of non-polar carbon. After TA introduction, the C1 content in BZT decreased to 56.44%, while the C2 content increased from 21.9% to 27.43%, confirming that the phenolic hydroxyl structure of TA has been successfully introduced to the surface. In BZTA, the C1 content further decreased to 51.57%, accompanied by an increase in C2 content to 34.75%. This trend indicates that the introduction of Ag^+^ not only preserved the TA structure but also further stabilized the surface oxygen-containing functional groups through coordination interactions.

The O1s spectra of BZ, BZT, and BZTA were deconvoluted into three components: O1 (lattice oxygen), O2 (oxygen vacancy), and O3 (adsorbed oxygen) [42]. As shown in Table 1, sample B mainly displayed O3 (93.93%), with minor amounts of O2 and negligible lattice oxygen. Upon ZnO loading (BZ), the O1 component notably increased to 33.41%, and O2 to 44.89%, confirming the establishment of a distinct ZnO lattice structure. Following the introduction of TA (BZT), both O1 and O2 rose further to 36.34% and 47.0%, respectively, indicating the inclusion of extra hydroxyl groups from TA and their bonding with the ZnO surface. In BZTA, both O1 and O2 slightly decreased to 31.41% and 39.0%, respectively. This decline is likely due to the competitive coordination between Ag^+^ and Zn^2+^, which might have partially modified the ZnO-TA-Ag composite structure on the bamboo surface, consistent with SEM observations. Additionally, some of the phenolic hydroxyl groups of TA were utilized in reducing Ag^+^, resulting in a reduction in exposed hydroxyl groups. Nonetheless, lattice oxygen remained the primary species, suggesting that the ZnO structure was predominantly maintained.

The Zn 2p spectra of BZ, BZT, and BZTA (Figure 4) exhibited features characteristic of ZnO. The Zn 2p_3/2_ binding energies were 1021.7–1022.0 eV, which is the standard value of wurtzite ZnO (1021.8 eV). No signal for Zn(OH)_2_ (1022.4 eV) was observed, indicating zinc was mostly Zn^2+^ in the ZnO lattice (XRD). The spin–orbit splitting energy was 23.0 eV on all samples. This confirms the stability of the ZnO crystal structure [43]. Notably, the presence of TA and Ag did not induce any noticeable lattice distortion. For sample BZTA, the Ag 3d spectrum (Figure 4d) exhibited two peaks at 367.85 eV (Ag 3d_5/2_) and 373.86 eV (Ag 3d_3/2_), with a spin–orbit splitting of 6.0 eV, indicative of metallic Ag(0) [44]. The absence of the peak at 368.4 eV, which is characteristic of Ag^+^, confirmed the complete reduction of the introduced Ag^+^ to elemental silver by TA.

### 2.4. Thermal Stability Analysis

**Figure 5:**
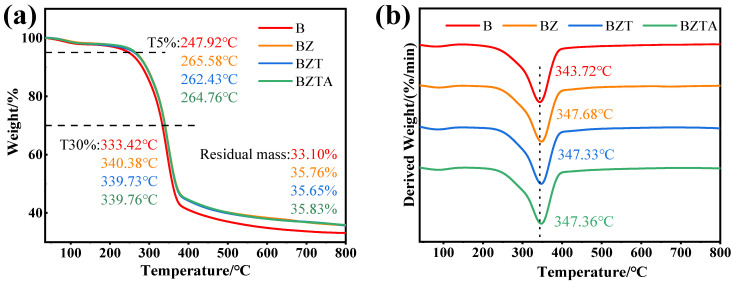
The TG (**a**) and DTG (**b**) curves of B, BZ, BZT, and BZTA.

**Figure 6:**
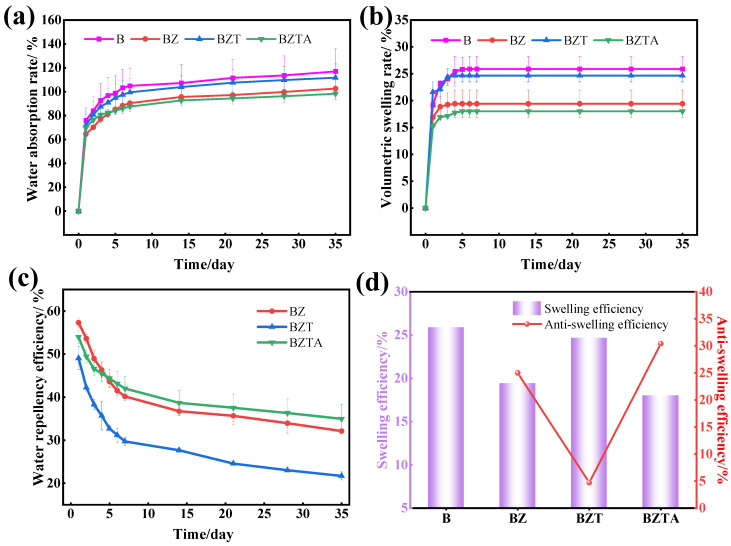
The water absorption rate (**a**), volume swelling rate (**b**), water repellency efficiency (**c**), swelling efficiency, and anti-swelling efficiency (**d**) of B, BZ, BZT, and BZTA.

The thermogravimetric (TG) curves for the modified bamboo samples are shown in Figure 5. The pyrolysis process of these modified bamboo samples can be broadly categorized into three stages. The first stage, from 30 °C to 150 °C, is attributed to the evaporation of physically adsorbed water in bamboo, with mass loss rates of 2.55%, 2.26%, 2.60%, and 2.27% being recorded for B, BZ, BZT, and BZTA, respectively, indicating slight variations in moisture absorption among the modified samples. The second stage, from 200 °C to 450 °C, is defined as the main pyrolysis interval, where significant weight loss was observed, primarily caused by the degradation or depolymerization of hemicellulose and cellulose, as well as the partial decomposition of lignin [45,46]. During this stage, the weight loss rate of the B sample was determined to be 58.49%, whereas those of the modified samples (BZ, BZT, and BZTA) were measured to be approximately 56%, slightly lower than that of B. The third stage (above 450 °C) is associated with the further degradation of lignin and the carbonization of cellulose and lignin residues [45]. At 800 °C, the residual mass values of B, BZ, BZT, and BZTA were found to be 33.10%, 35.76%, 35.65%, and 35.83%, respectively, indicating that the incorporation of ZnO and its composites enhanced the high-temperature residual mass of bamboo. The enhanced thermal stability of modified samples is attributed to the catalytic effect of ZnO. ZnO acts as a Lewis acid catalyst that promotes cellulose dehydration over depolymerization, shifting pyrolysis toward a char-forming pathway rather than a volatile-releasing pathway [47]. Additionally, ZnO, ZnO-TA, and ZnO-TA-Ag nanoparticles serve as a physical barrier, hindering volatile escape and promoting secondary char formation. From the corresponding derivative thermogravimetric (DTG) curves (Figure 6b), it was observed that the temperatures corresponding to the maximum weight loss rates (T_max_) for all samples ranged from 343 °C to 348 °C, following the order B < BZ ≈ BZT ≈ BZTA. This suggests that the thermal stability of bamboo was improved by the introduction of ZnO and its composites, while no significant alteration was caused by the subsequent addition of TA and silver ions.

### 2.5. Analysis of Dimensional Stability

The water absorption rate, volume expansion rate, water resistance efficiency (WRE), and anti-swelling efficiency (ASE) of B, BZ, BZT, and BZTA, as functions of time, are presented in Figure 6. As shown in Figure 6a, the water absorption and volume expansion of all samples exhibited similar trends over time. During the initial soaking period (≤7 days), water absorption rates increased rapidly before gradually stabilizing. After 35 days, the WRE values of B, BZ, BZT, and BZTA were 151.07%, 102.55%, 118.29%, and 98.22%, respectively. The B sample exhibited a significantly higher water absorption rate than the three modified samples, with BZTA having the lowest value. This suggests that surface modification reduces the water uptake of bamboo. Figure 6b shows that the volume swelling rates for all samples increased strongly in the first 5 days, before plateauing. After 35 days, the volume swelling rates for B, BZ, BZT, and BZTA were 25.88%, 19.41%, 24.66%, and 18.01%, respectively, following the order of B > BZT > BZ > BZTA. This trend is consistent with that found for WRE, indicating that the modification treatment not only inhibits the water absorption of bamboo but also significantly reduces its volume expansion, while improving its dimensional stability. As can be seen in Figure 6c, the BZTA sample has better WRE, reaching 34.98% at 35 days. The BZ sample comes next with a WRE of 32.12%, while the WRE of the BZT sample is relatively low, standing at 21.70%. Regarding anti-swelling efficiency (ASE), as shown in Figure 6d, the ASE values of BZ, BZT, and BZTA were 24.98%, 4.73%, and 30.41%, respectively. Remarkably, the ASE value of BZTA was approximately 1.2 times and 6.4 times greater than those of BZ and BZT, respectively. This demonstrates that BZTA is the best at improving the water resistance and dimensional stability of bamboo.

The water resistance and dimensional stability of bamboo depend on its swelling behavior at water absorption equilibrium and the presence of hydrophilic groups on its surface [48]. Previous research has shown that water adsorption necessitates adequate adsorption sites and accommodation space, predominantly influenced by the quantity and accessibility of hydroxyl groups in bamboo, along with its pore structure [49]. In this study, ZnO was synthesized in situ on the bamboo surface through interaction with cellulose hydroxyl groups, thereby masking a portion of the hydrophilic sites. Further, as shown in SEM images, ZnO particles are filled in the fiber gaps and cavities of bamboo, thereby reducing its porosity. This reduction limits the moisture adsorption sites and pathways, leading to a decline in the water absorption rate and volume expansion rate. The BZT samples showed slightly higher WRE and VSR than BZ, likely because TA introduces phenolic hydroxyl groups that increase surface hydrophilicity. By contrast, BZTA exhibited improved water resistance, probably because TA’s hydroxyl groups are partially consumed during Ag^+^ reduction, leaving fewer hydrophilic sites and further suppressing moisture uptake. In summary, incorporation of ZnO, ZnO-TA, and ZnO-TA-Ag all decreases bamboo’s WAR and VSR, enhancing its moisture resistance and dimensional stability. Among these, the BZTA sample delivered the best overall performance.

### 2.6. Microstructure Analysis

**Figure 7:**
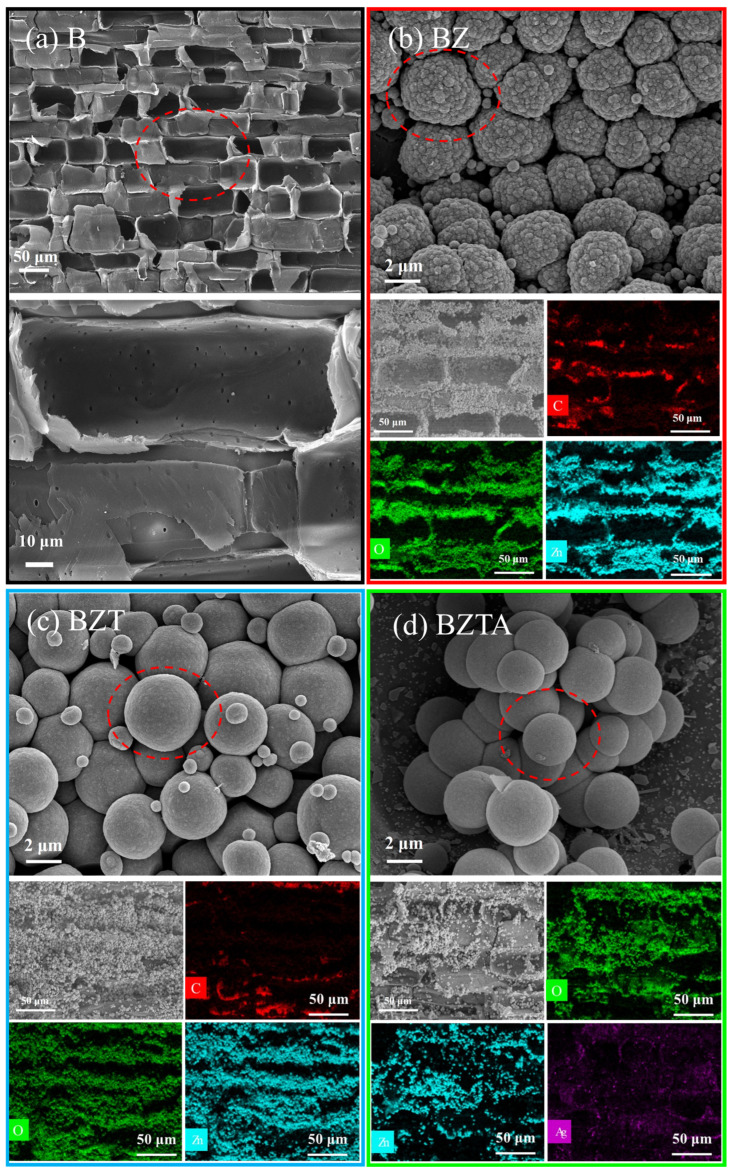
SEM images, EDS mappings, and spectra of B (**a**), BZ (**b**), BZT (**c**), and BZTA (**d**).

The SEM and EDS spectra of various bamboo samples are illustrated in Figure 7. The surface of the parenchyma cell layer in the original bamboo appears notably smooth with numerous natural pits in the cavity. Subsequent modification treatments reveal distinct morphologies of particles on the surfaces of BZ, BZT, and BZTA samples in the SEM images. The particles observed on the BZ sample resemble miniature broccoli, forming a “flower-ball-shaped” aggregate structure. Conversely, in the BZT and BZTA samples, featuring the addition of TA or TA-Ag^+^, the particle surfaces exhibit a smoother texture, leading to the disappearance of the flower-ball-shaped structure. This observation suggests that the incorporation of TA induces alterations in the surface morphology and roughness of the composite particles, significantly influencing their assembly behavior. Various treatment methods lead to varying particle loading levels on sample surfaces. Specifically, the cell cavities of the BZ and BZT samples are almost completely filled with particles, whereas the particle content in the BZTA sample is comparatively lower. This observation aligns with the bamboo weight gain rate outcomes (Figure 1). The EDS analysis results indicate that the primary elements of the BZ, BZT, and BZTA samples are C, O, and Zn, with a minor presence of silver in the BZAT sample. Corroborated by XRD and FT-IR findings, these observations verified the successful deposition of ZnO, ZnO-TA complexes, and ZnO-TA-Ag composite particles on BZ, BZT, and BZTA, respectively.

### 2.7. Antimildew Properties

**Figure 8:**
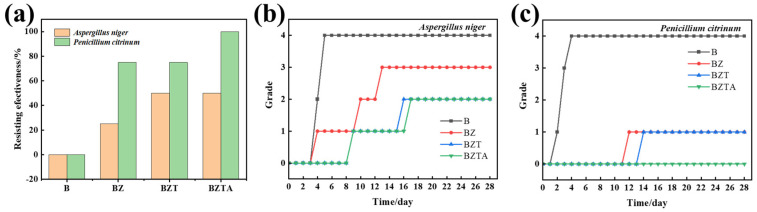
The resisting effectiveness (**a**) and grades of the bamboo samples against *Aspergillus niger* (**b**) and *Penicillium citrinum* (**c**).

**Figure 9:**
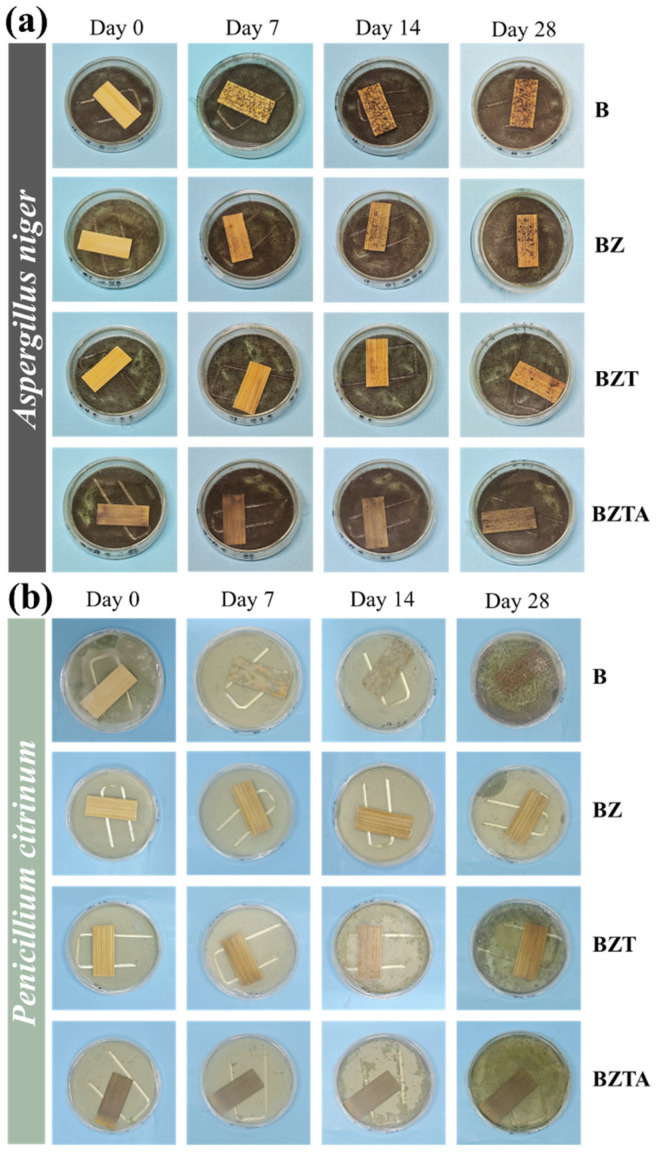
Photos of the bamboo samples during testing against *Aspergillus niger* (**a**) and *Penicillium citrinum* (**b**).

Figure 8 and Figure 9 show the antimildew performance and test photographs of the B, BZ, BZT, and BZTA bamboo samples against *Aspergillus niger* and *Penicillium citrinum*. As shown in Figure 8b,c, natural bamboo B exhibited the fastest infection progression, with infected areas exceeding 75% coverage by day 5 for *Aspergillus niger* and by day 4 for *Penicillium citrinum*, reaching the maximum infection level of grade 4. By contrast, the ZnO-loaded BZ samples showed only grade 1 infection by *Aspergillus niger* and *Penicillium citrinum* on day 4 and day 12, respectively. On BZ, *Aspergillus niger* infection remained at grade 1 through to day 9; by day 28, it exceeded 50% coverage and rose to grade 3, whereas *Penicillium citrinum* remained at grade 1. These results indicate that ZnO loading delays bamboo decay but provides limited antifungal efficacy. For the BZT sample, *Aspergillus niger* and *Penicillium citrinum* first produced grade 1 infections on days 9 and 14, respectively.

With extended incubation, the BZT sample reached grade 2 infection by *Aspergillus niger* on day 16 and remained at that level, while *Penicillium citrinum* infection stayed stable at grade 1. The BZTA samples showed *Aspergillus niger* infection beginning on day 9, matching BZT at grade 1. BZTA reached grade 2 one day later than BZT (on day 17) and remained at that level thereafter. Although both BZT and BZTA ultimately exhibited the same infection grade for *Aspergillus niger*, the infected surface area on BZT was approximately 50%, compared with about 40% on BZTA, indicating superior mold resistance of BZTA to *Aspergillus niger*. Throughout the entire 28-day incubation period, BZTA completely resisted *Penicillium citrinum* infection, showing no mold growth on its surface and an infection area of 0 (Figure 9b), which demonstrates superior resistance to *Penicillium citrinum*.

It is evident that the loading of ZnO-TA-Ag particles effectively enhances the mold resistance of bamboo. The anti-mold action of BZTA samples is due to two main reasons: Firstly, ZnO-TA-Ag particles fill bamboo cell cavities and occupy hydrophilic sites on the surface, which improves material hydrophobicity and makes it difficult for fungal adhesion and growth to take place. Secondly, there is a chemical inhibition effect, where long-term release of Zn^2+^ and Ag^+^ particles combined with TA in BZTA samples produces a synergistic antibacterial effect, disrupting fungal cell membrane structure, inhibiting hyphal growth and spore growth, leading to a stronger antibacterial effect. In summary, all modified bamboo materials demonstrated varying degrees of resistance to mold, and BZTA samples loaded with ZnO-TA-Ag composite particles exhibited the most superior overall antifungal efficacy against both *Aspergillus niger* and *Penicillium citrinum*.

## 3. Materials and Methods

### 3.1. Materials and Chemicals

Three-year-old Bamboo (*Dendrocalamus giganteus*), devoid of mildew and pest damage, was obtained in Yunnan Province, China. After removing the outer epidermal and inner parenchyma layers, the bamboo was cut into specimens measuring 50 mm × 20 mm × 2 mm for subsequent experiments. TA and Zinc acetate (Zn(CH_3_COOH)_2_) were provided by Aladdin Industrial Corporation (Shanghai, China). Folin–Ciocalteu and potato dextrose agar (PDA) were purchased from Macklin (Shanghai, China). Silver nitrate (AgNO_3_) and ethanol were obtained from Ghtech (Guangzhou, China). All chemicals were of analytical grade and used as received.

### 3.2. Preparation of ZnO-TA-Ag Composite Bamboo

**Figure 10:**
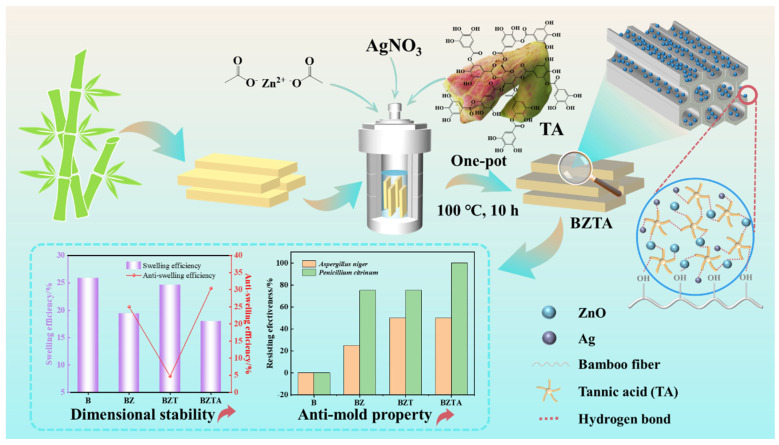
Schematic diagram of constructing ZnO-TA-Ag composite bamboo.

ZnO, ZnO-TA and ZnO-TA-Ag composite Bamboo were synthesized via a hydrothermal reaction (Figure 10). For the synthesis of ZnO-modified bamboo (BZ), zinc acetate (1.0 g) was dissolved in 40 mL of ethanol in a 100 mL hydrothermal reactor. A predetermined amount of bamboo substrate was then immersed in the solution, after which the reactor was sealed and maintained at 100 °C for 10 h. Following natural cooling to room temperature, the bamboo samples were retrieved and sequentially washed with ethanol and deionized water (three times each), before being air-dried at room temperature for 6 h and, finally, oven-dried at 60 °C for 12 h. After removal, the samples were cooled to room temperature in a desiccator, weighed, and stored for subsequent use. The bamboo–ZnO–TA (BZT) composite was prepared using a similar procedure, with the addition of 0.05 g of tannic acid (TA) to the initial zinc acetate solution. For the bamboo–ZnO–TA–Ag (BZTA) composite, both 0.05 g of TA and 0.05 g of silver nitrate were introduced into the precursor solution.

The effects of hydrothermal reaction temperature (80, 90, 100, 110, 120 °C), time (1, 2, 4, 6, 8, 10, 12 h), and the mass concentration ratio of zinc acetate to tannic acid (40:1, 30:1, 20:1, 15:1, 10:1) on the loading amount of TA and composite particles on the bamboo surface were investigated.

### 3.3. Quantification of TA on the Bamboo Surface Using the Folin–Ciocalteu Method

The Folin–Ciocalteu (FC) method was employed to determine the total phenolic content on the surface of the functionalized bamboo samples [50,51]. Briefly, the samples were immersed in a mixture of 8 mL distilled water and 0.5 mL FC for 10 min, followed by the addition of 1.5 mL of sodium carbonate solution (Na_2_CO_3_, 7.5% *w*/*v*). After incubation at room temperature for 2 h, the absorbance of the resulting solution was measured at 765 nm using a Microplate Reader (Thermo Scientific, Waltham, MA, USA). A calibration curve was constructed using gallic acid (GA) as a standard, and the results were expressed as μg of GA equivalents per square centimeter (μg GA eq/cm^2^) of bamboo surface area.

### 3.4. Characterizations

The chemical structure of the sample’s surface before and after treatment was characterized by X-ray photoelectron spectroscopy (XPS) (K-Alpha, Thermo Scientific, USA) and Fourier-transform infrared (FTIR) spectroscopy (Tenson 27, Bruker, Ettlingen, Germany). FTIR spectra were acquired in ATR mode over a range spanning 4000 to 400 cm^−1^ at a resolution of 4 cm^−1^ and 32 scans. X-ray diffraction (XRD) analysis was performed on a multifunctional X-ray diffractometer (Ultima IV, Rigaku, Tokyo, Japan) over a 2θ range of 5–80° at a scanning rate of 5°⋅min^−1^. Surface morphology and elemental mapping were examined using scanning electron microscopy (SEM) (ZEISS Sigma 300, Oberkochen, Germany), coupled with X-ray energy dispersive spectrometry (EDS). Prior to observation, the samples were sputter-coated with platinum to enhance conductivity, and the accelerated voltages for imaging and EDS analysis were 5 kV and 15 kV, respectively. Thermogravimetric analysis (TGA) was conducted on a thermogravimetric analyzer (TG 209 FC, Netzsch, Selb, Germany) by heating from 30 °C to 800 °C at a rate of 10° min^−1^ under a nitrogen atmosphere.

### 3.5. Physical Properties of Bamboo

The bamboo samples were immersed in deionized water, kept away from light, and weighed; we also measured their dimensions at different time intervals (1, 2, 3, 4, 5, 6, 7, 14, 21, 28, and 35 days). The water absorption rate (WAR) and volume swelling rate (VSR) were calculated as follows [52]:

(1) $$WAR=\frac{\left(m_{i}-m_{0}\right)}{m_{0}}\times 100\%$$

(2) $$VSR=\frac{\left(V_{i}-V_{0}\right)}{V_{0}}\times 100\%$$

where *m*_0_ and *V*_0_ are the mass and volume of the bamboo before impregnation, respectively; *m_i_* and *V_i_* are the mass and volume of the bamboo after impregnation on day 1, respectively.

The water resistance and dimensional stability of the bamboo samples were evaluated using water resistance efficiency (WRE) and anti-swelling efficiency (ASE), calculated as follows [52]:

(3) $$WRE=\frac{{WAR}_{C}-{WAR}_{i}}{{WAR}_{C}}\times 100\%$$

(4) $$SE=\frac{\left(V_{t}-V_{0}\right)}{V_{0}}\times 100\%$$

(5) $$ASE=\frac{{SE}_{c}-{SE}_{t}}{{SE}_{C}}\times 100\%$$

where *WAR_C_* represents the water absorption rate of the control group (untreated bamboo B) after being impregnation for 35 days, and *WAR_i_* is the water absorption rate of the sample after 1 day of immersion; *V_t_* is the volume of the bamboo samples after 35 days of immersion, while *SE_C_* and *SE_t_* represent the volume expansion rate of the control group and the treated samples after 35 days of immersion, respectively.

### 3.6. Test of Mildew Resistance

The antimildew performance was evaluated according to the Chinese National Standard [GB/T 18261-2013] [53]. *Aspergillus niger* and *Trichoderma viride* were selected for antimildew testing. The detailed procedures have been reported previously [9,35]. Mildew coverage on the sample surfaces was classified into five grades: 0 (0%), 1 (0–25%), 2 (25–50%), 3 (50–75%), and 4 (70–100%). The mildew-resisting effectiveness (*RE*) against the two fungal strains was then determined by Equation (6):

(6) $$RE=\left(1-\frac{D_{1}}{D_{0}}\right)\times 100\%$$

where *RE* represents the comprehensive resisting effectiveness, and *D*_1_ and *D*_0_ correspond to the infection values of the treated and untreated samples, respectively.

## 4. Conclusions

In this study, we developed a one-step hydrothermal modification method mediated by TA to functionalize bamboo surfaces, thereby overcoming the limitations of traditional two-step processes and enabling simultaneous functionalization. Under optimal process conditions, we demonstrated that the BZTA composites improved WAR by 52.85% and increased ASE to 30.41%. Furthermore, antimildew activity for *Aspergillus niger* and *Penicillium citrinum* increased from grade 4 to grades 1 and 0. This performance improvement may be attributed to the combined effect of the physical barrier formed by the in situ ZnO/TA/Ag layer and the antibacterial properties of the composite particles. This modified approach eliminates harmful organic antifungal agents and also simplifies the loading of functional additives on the substrate, thereby making it a sustainable and ecologically sustainable method of bamboo enhancement. This novel method provides a feasible pathway for producing high-performance multifunctional bamboo materials and provides a reference for organic–inorganic hybrid nanomaterials in biomass materials.

## Figures and Tables

**Table 1:** C1s division peak percentage contents and O/C ratios of samples.

Samples	Carbon Components				O Components			O/C (%)
	C1/%	C2/%	C3/%	C4/%	O1/%	O2/%	O3/%	
B	47.15	39.01	9.71	4.12		6.07	93.93	48.70
BZ	65.16	21.90	11.45	1.49	33.41	44.89	21.70	80.31
BZT	56.44	27.43	7.30	8.83	36.34	47.00	16.66	102.90
BZTA	51.57	34.75	9.77	3.90	31.41	39.00	29.59	86.22

## Data Availability

Data are contained within the article and Supplementary Materials.

## Supplementary Materials

The following supporting information can be downloaded at: https://www.mdpi.com/article/10.3390/molecules31101737/s1. Figure S1. SEM of the bamboo samples (a) BZ4, (b) BZ12, (c) BZT4, (d) BZT12, (e) BZTA4, (f) BZTA12. Figure S2. FTIR spectra of BZ4, BZT4, and BZTA4. Figure S3. FTIR spectra of BZ12, BZT12, and BZTA12. Table S1. ATR-FTIR absorbance intensity ratios of BZT and BZTA. Figure S4. Effect of pH on the TA content of BZT and BZTA samples after stability test.
