# Supplementary material for: Simultaneous Enhancement of Mildew Resistance and Dimensional Stability of Bamboo with a Facile One-Step In Situ Growth of ZnO/TA/Ag Composites
Source: Molecules. 2026 May 19;31(10):1737. doi: 10.3390/molecules31101737 (PMC13209443; doi:10.3390/molecules31101737)
Supplement: Supplementary file 1 [file molecules-31-01737-s001.zip › molecules-4309144-supplementary.pdf]

Simultaneous enhancement of mildew resistance and dimensional stability of bamboo with a facile one-step *in situ* growth of ZnO/TA/Ag composites

Juan Xu[1,2,3], Jinju Ma[2,3], Lanxiang Liu[2,3], Baoshan Tang[2,3], Hong Zhang[2,*], Wenwen Zhang[4], Zhengjun Shi[1,*]

1 Key Laboratory of State Forestry and Grassland Administration on Highly-Efficient Utilization of Forestry Biomass Resources in Southwest China, Southwest Forestry University, Kunming 650224, China; xujuan89@hotmail.com (J.X.)

2 Institute of Highland Forest Science, Chinese Academy of Forestry, Kunming 650233, China; lanxiangliu@outlook.com (L.L.); majinju1231km@hotmail.com (J.M.); tangbaos@163.com (B.T.)

3 Yunnan Key Laboratory of Breeding and Utilization of Resource Insects, Kunming 650224, China;

4 School of Pharmacy, Xinyang Agriculture and Forestry University, Xinyang 464000, China; zhangwenwen1105@163.com (W.Z.)

* Correspondence: shizhengjun1979@swfu.edu.cn (Z.S.); kmzhh@hotmail.com (H.Z.)

1. SEM of Samples (BZ, BZT, BZTA) Prepared at 4 h and 12 h

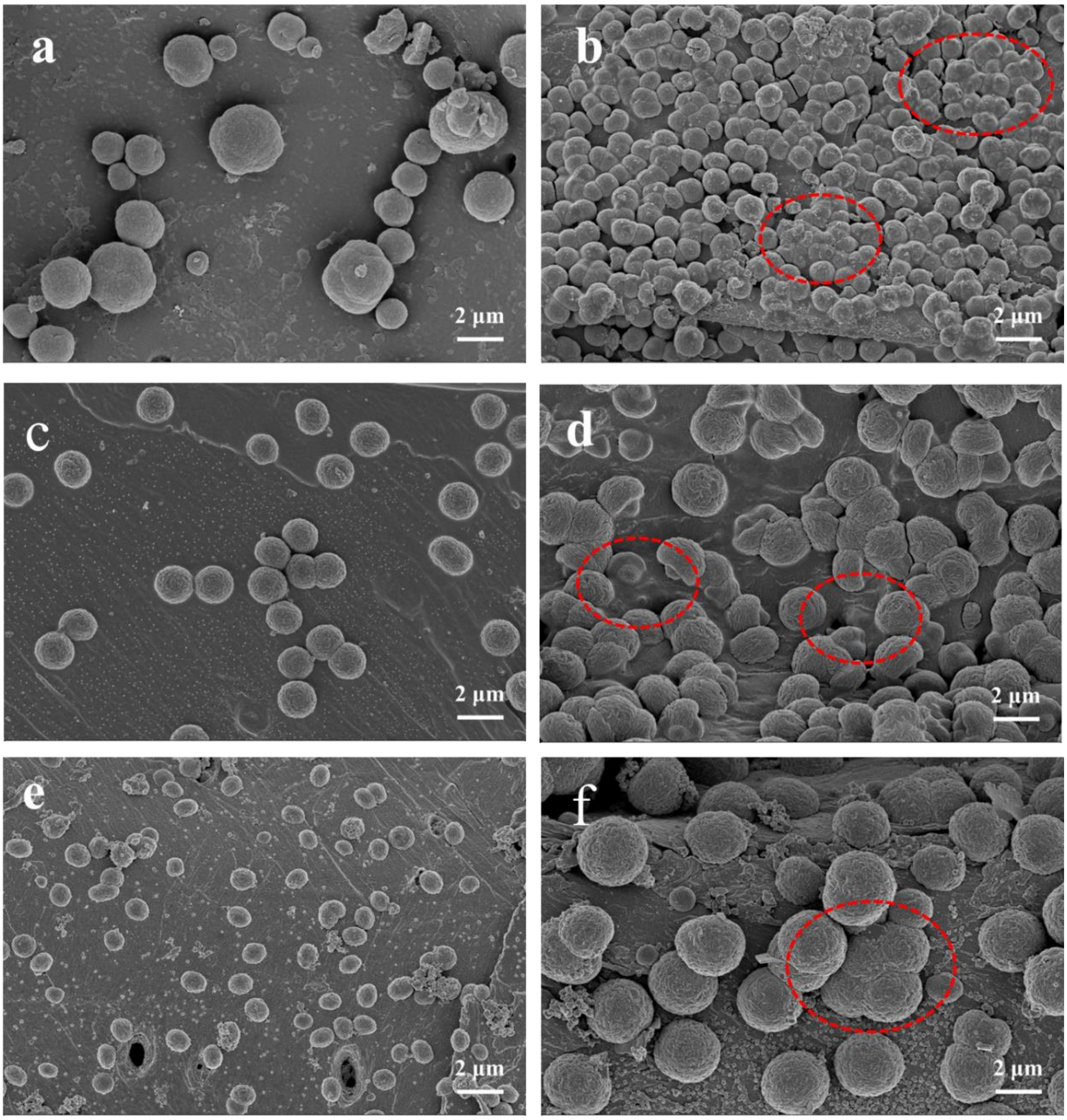

Figure S1. SEM of the bamboo samples (a) BZ_4, (b) BZ_{12}, (c) BZT_4, (d) BZT_{12}, (e) $BZTA_4$, (f) $BZTA_{12}$.

2. FTIR Spectra of Samples (BZ, BZT, BZTA) Prepared at 4 h and 12 h

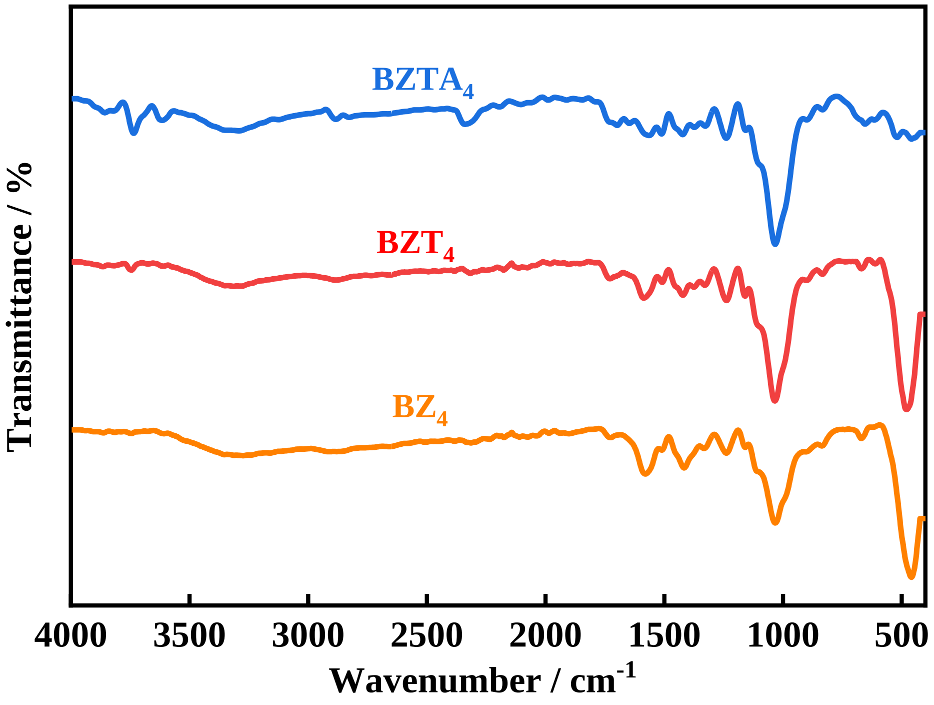

Figure S2. FTIR spectra of BZ_4, BZT_4, and $BZTA_4$.

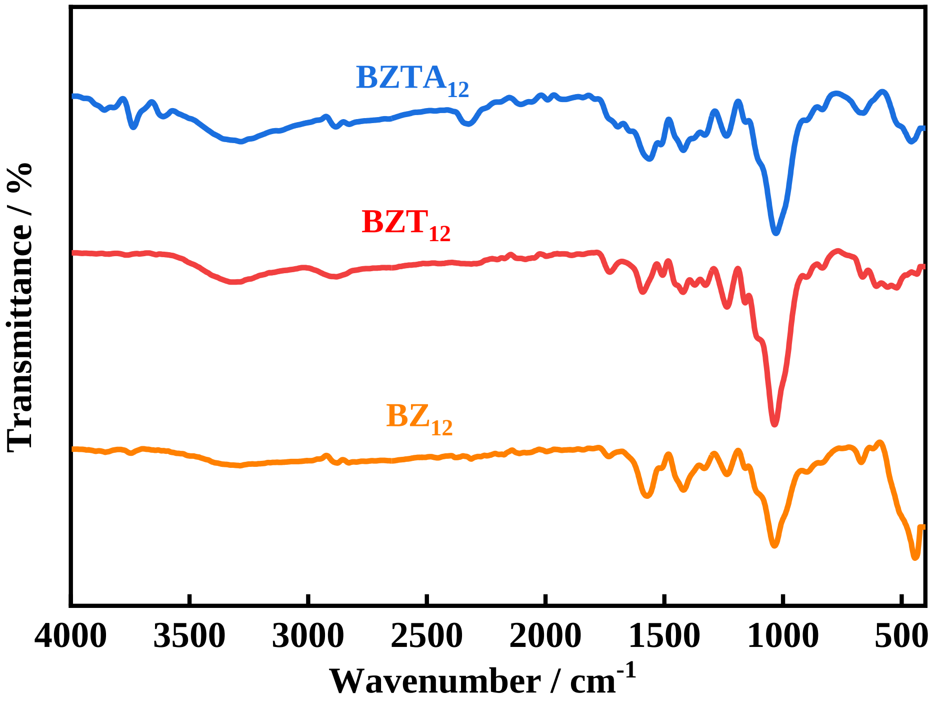

Figure S3. FTIR spectra of BZ_{12}, BZT_{12}, and $BZTA_{12}$.

Table S1. ATR-FTIR absorbance intensity ratios of BZT and BZTA.

	BZT_4	BZT_{10}	BZT_{12}	$BZTA_4$	$BZTA_{10}$	$BZTA_{12}$
I_{3340}/I_{1725}	1.50	4.60	1.38	1.56	6.67	1.95
I_{1240}/I_{1725}	2.86	5.50	2.52	2.50	6.33	2.05

Note: I_{3340}, I_{1725} and I_{1240} represent the peak intensities at 3340 cm^{-1}(OH), 1725 cm^{-1}(C=O) and 1240 cm^{-1}(C-O) respectively. BZT_n and $BZTA_n$ denote the BZT and BZTA samples obtained after a reaction time of n hours, respectively.

3. Stability test

Samples (BZT and BZTA) were hydrothermally treated in 40 mL of buffer solutions (0.01 mol/L, pH 4 , 7, and 9) at 100 °C for 2 h. After cooling naturally, the samples were rinsed with deionized water until colorless and dried. The phenolic content remaining on the substrates was measured via the Folin–Ciocalteu colorimetric assay. The result of the stability test is shown in Figure S4.

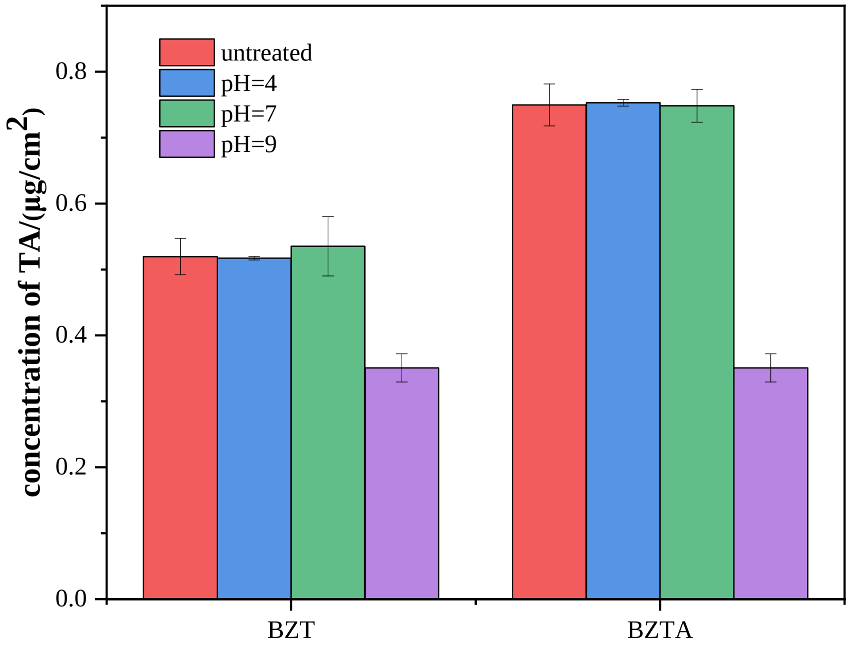

Figure S4. Effect of pH on the TA content of BZT and BZTA samples after stability test.
